# Distributed environmental sensing and soil moisture gradient navigation in modular robots

**DOI:** 10.3389/frobt.2026.1895019

**Published:** 2026-08-24

**Authors:** Henry Alberto Hernandez Martinez, Holman Montiel Ariza, Luis Fernando Pedraza Martinez

**Affiliations:** 1 Doctoral Program in Engineering, Faculty of Engineering, Universidad Distrital Francisco José de Caldas, Bogotá, Colombia; 2 Faculty of Technology, Universidad Distrital Francisco José de Caldas, Bogotá, Colombia

**Keywords:** agricultural robotics, autonomous exploration, central pattern generators, chain-type robots, cooperative decision-making, directional estimation, distributed control, environmental monitoring

## Abstract

Agricultural environments exhibit spatial variations in soil moisture that provide directional information for exploration and monitoring tasks. This work presents a distributed navigation strategy for modular robots based on local soil moisture gradients and neighbor-to-neighbor information exchange. The proposed architecture separates navigation and locomotion into two coordinated layers. The navigation layer estimates movement direction from environmental measurements and obstacle perception. The locomotion layer transforms directional decisions into coordinated motion through coupled oscillators. The proposed method was implemented on an EMeRGe modular robotic platform and was evaluated through 360 physical experiments conducted with three modular configurations under heterogeneous soil moisture conditions. The experimental evaluation compared the proposed method with Hill Climbing, Ant Colony Optimization, and Genetic Algorithm navigation strategies operating under identical sensing and locomotion conditions. The obtained results showed reductions in convergence time, decreases in directional estimation error, increases in navigation efficiency, and lower velocity variance across the evaluated scenarios. These results indicate that local soil moisture gradients can be used to estimate navigation directions without global maps or centralized control. This approach establishes a relationship between environmental sensing, distributed directional estimation, and motion generation in modular robotic systems for agricultural monitoring applications.

## 1 Introduction

Agricultural environments present spatial heterogeneity in soil properties that affects irrigation management, crop development, and field operations. Soil moisture is used to characterize this heterogeneity because it influences water availability, nutrient transport, root development, and soil trafficability. Agricultural soils usually exhibit volumetric water content values between 10% and 40%, depending on irrigation practices, soil composition, and climatic conditions. These spatial differences occur at scales between 0.5 m and 10 m, generating heterogeneous regions within the same field (Food and Agriculture Organization of the United Nations, 2006; Krueger et al., 2024).

Precision agriculture requires measurements with adequate spatial resolution to identify such field variability. Conventional monitoring procedures commonly rely on manual sampling or fixed sensing stations. Manual sampling provides data at discrete locations and requires human intervention, whereas fixed stations offer continuous information only at specific points. Consequently, both approaches may fail to capture changes occurring between sampling locations (Moshayedi et al., 2024). Mobile robotic systems constitute an alternative for acquiring field information with higher spatial and temporal sampling density (Pincheira et al., 2023).

Despite these advances, several agricultural robotic approaches still depend on predefined trajectories, global maps, external localization systems, centralized processing, or navigation strategies that do not directly use *in situ* measurements for motion decisions. This separation limits the ability of the robot to convert soil moisture patterns into motion decisions during operation. In monitoring tasks involving heterogeneous soil conditions, the robot should not only move through the environment but also use the measured field as a directional cue for exploration (Martínez et al., 2023; Hernández et al., 2025).

Modular robotic systems provide a physical structure for acquiring measurements at different positions of the robot and exchanging information among neighboring modules. This configuration is relevant for soil moisture monitoring because directional decisions can be derived from local field variations without requiring a complete global map. However, existing approaches still provide limited integration between soil moisture sensing, distributed directional estimation, and physical locomotion, since navigation decisions are frequently produced by planning, optimization, or predefined rules rather than by the measured field itself (Liu et al., 2023; Lee et al., 2024; Lancheros et al., 2025; Hernández et al., 2025).

These limitations motivate the development of navigation strategies that use local field information while preserving distributed control. In this work, the navigation problem is formulated as the estimation of a movement direction from measurements of a soil moisture field acquired through distributed sensing and exchanged among neighboring modules. In this context, *exploration* refers to the process of navigating the environment while collecting soil moisture information; navigation determines the direction of motion, whereas sampling acquires field measurements during displacement. Based on this formulation, a distributed navigation strategy for modular robots is proposed using soil moisture gradients and neighbor-to-neighbor information exchange. The strategy is referred to as Soil-Moisture Gradient Distributed Navigation (SMG-DN). SMG-DN transforms field measurements into directional decisions without relying on global maps, centralized controllers, or iterative search procedures. In the experimental implementation, soil moisture is estimated from calibrated infrared reflectance measurements, whereas obstacle perception is obtained from proximity sensors distributed on the module faces. The control architecture is organized into a navigation layer that estimates the desired direction of motion and a locomotion layer that transforms this estimate into coordinated physical displacement through coupled oscillators. The contributions of this work are summarized as follows:

1. A distributed navigation controller that estimates movement direction from soil moisture gradients through neighbor-to-neighbor information exchange.
2. A two-layer control architecture that separates directional estimation from locomotion generation.
3. An integration mechanism between distributed environmental sensing and CPG-based locomotion.
4. A comparative evaluation against Hill Climbing, Ant Colony Optimization, and Genetic Algorithm navigation strategies under identical sensing and locomotion conditions.
5. A physical validation using a modular robotic platform operating under heterogeneous soil moisture conditions.

The remainder of the article is organized as follows. Section 2 reviews the literature related to distributed robotic systems, environmental navigation, and locomotion control. Section 3 presents the proposed control architecture, including the problem formulation, the distributed navigation layer, and the CPG-based locomotion layer. Section 4 describes the robotic platform, the baseline algorithms used for comparative evaluation, and the experimental protocol used for validation. Section 5 presents the experimental results.

## 2 Related work

Agricultural monitoring has motivated the development of robotic systems for collecting field data under spatially heterogeneous soil conditions. Agricultural platforms can be classified according to mobility mechanism, sensing configuration, and control architecture. Wheeled and tracked robots are used for ground inspection, transportation, and in-field monitoring, whereas aerial vehicles provide coverage of large areas through remote sensing (Zhang et al., 2023; Hernández et al., 2025). These platforms support precision agriculture; however, their motion planning strategies often depend on predefined routes, external localization, global maps, or centralized processing. Therefore, the direct use of soil moisture variations as a navigation cue remains limited in agricultural robotic applications.

Modular robotic systems provide an alternative framework for distributed sensing and cooperative control. In these systems, sensing, actuation, communication, and processing capabilities are distributed across interconnected modules. This configuration allows measurements to be acquired at multiple locations along the robotic body while preserving information exchange among neighboring units (Tian and Zhan, 2021; Liu et al., 2023; Stroppa et al., 2023). Several modular platforms have focused on structural reconfiguration to adapt morphology according to task requirements or environmental constraints (Nisser et al., 2022; Zhao et al., 2023). However, reconfiguration alone does not specify how field measurements are converted into directional decisions. For agricultural monitoring, this distinction is relevant because the robot must not only adapt its morphology or gait pattern but also use local soil conditions to determine where to move.

Control architecture plays a central role in the conversion of sensed data into motion commands. Centralized architectures process information through a single controller with access to global or aggregated system states. Decentralized approaches distribute sensing, communication, and decision-making among individual agents or modules. Hybrid schemes combine global planning with local adaptation mechanisms (Moreno and Faiña, 2021; Ju and Son, 2021; Mu et al., 2025). In heterogeneous agricultural environments, distributed architectures are relevant because they allow measurements acquired at different body locations to influence motion without requiring complete knowledge of the field. Nevertheless, coupling distributed perception with coordinated physical displacement remains an open problem in modular robotics.

Navigation and locomotion correspond to related but distinct functions within robotic exploration. Navigation determines the desired direction from available information, whereas locomotion produces the physical displacement required to execute that decision. This distinction is important in modular robots because a directional command must be translated into coordinated actuator trajectories across multiple units. Central Pattern Generator (CPG) models have been employed for this purpose because they generate rhythmic motion through coupled oscillators that regulate phase, amplitude, and frequency relationships among actuators (Sun et al., 2021). Several studies have applied CPG-based controllers to modular, snake-like, and articulated robots operating under distributed or partially distributed schemes (Zhao et al., 2024; Liu et al., 2024). However, in many of these approaches, rhythmic motion is generated independently of field sensing, and the directional reference is provided by an external planner, a predefined rule, or an operator-defined command.

Optimization-based methods constitute another group of approaches used in robotic exploration and path planning. Particle Swarm Optimization, Genetic Algorithms, Ant Colony Optimization, and related methods select candidate movements through objective functions that evaluate performance criteria (Sun et al., 2022; Shan et al., 2024; Liu and Wang, 2023). These methods are useful when the problem can be formulated as the minimization of a cost or the maximization of a utility function. However, their effectiveness depends on the sensed variables used to construct the objective function and on the computational resources required to evaluate candidate solutions. When navigation depends on physical terrain conditions, soil or obstacle-related variables must be explicitly included in the decision process (Roberts and Kim, 2023; Gupta and Zhang, 2024; Lee et al., 2024). In real-time modular robots, the computational cost of iterative or population-based methods may also limit their direct implementation within short control cycles.

Biologically inspired approaches have also been used to estimate motion direction from spatial field variations. The Bacterial Foraging Optimization Algorithm models agent displacement according to concentration changes in the environment and has been applied to optimization, path planning, and parameter estimation problems (Afonso et al., 2025; Nandini and Rahimunnisa, 2025). Several variants incorporate information exchange among agents, enabling collective or distributed decision-making mechanisms (Benjamin et al., 2023; Meng et al., 2025; Polaske et al., 2022; Lin et al., 2024; De Baets et al., 2025). These approaches are conceptually related to gradient-following behavior because displacement can be guided by local changes in a scalar field. Nevertheless, their application to physical modular robots using soil moisture gradients as the primary navigation signal remains limited.

Table 1 summarizes representative studies according to robot type, field cue, directional estimation method, distributed control, CPG-based locomotion, and physical validation. The comparison shows that previous studies address distributed robotic architectures, terrain-aware navigation, and motion generation as separate research problems. Some works focus on modular design or reconfiguration without explicitly incorporating environmental gradients into the navigation process. Other works use optimization or planning strategies but do not integrate distributed sensing with CPG-based locomotion. Similarly, several CPG-based approaches generate coordinated movement, although the directional reference is commonly defined outside the sensing loop.

**TABLE 1:** Comparison of representative studies on modular robotics, navigation, sensing, and locomotion.

References	Robot	Field cue	Direction method	Dist	CPG	Phys
Wirkus et al. (2020)	Modular	No	Rule-based	Yes	No	Yes
Moreno and Faiña (2021)	Modular	No	External	Yes	No	Yes
Liu et al. (2023)	Modular	No	Planning	Yes	No	Yes
Parada et al. (2021)	Modular	No	Reconfig	Yes	No	Yes
Tian and Zhan (2021)	Modular	No	Not addressed	Yes	No	Yes
Zhao et al. (2025)	Snake-like modular	No	RL	No	No	No
Shamshiri et al. (2024)	Agricultural robot	Local sensing	Assisted	No	No	Yes
Lee et al. (2024)	Mobile robot	Potential field	Optimization	No	No	Yes
Sun et al. (2022)	Modular robot	Env. Cost	GA	No	No	No
Shan et al. (2024)	Mobile robot	Obstacle map	ACO	No	No	No
Liu and Wang (2023)	Agricultural robot	Field data	ACO	No	No	No
Sun et al. (2021)	Legged robot	Sensory feedback	Not addressed	Yes	Yes	No
Liu et al. (2024)	Snake robot	No	External	No	Yes	Yes
Zhao et al. (2024)	Pneumatic robot	No	External	No	Yes	Yes
This work	Modular robot	Soil moisture gradient	Distributed gradient	Yes	Yes	Yes

Dist., distributed control; CPG, Central Pattern Generator locomotion; Phys., physical validation; RL, reinforcement learning; GA, Genetic Algorithm; ACO, Ant Colony Optimization.

The reviewed literature reveals a gap in the integration of environmental perception, distributed directional estimation, and locomotion generation within a unified control framework. Few studies formulate navigation as a distributed directional estimation problem driven by local soil moisture gradients while simultaneously coupling this decision process with coordinated locomotion. Experimental validation under heterogeneous soil conditions using physical modular robotic systems is also limited (Adetunji et al., 2022; Zhao et al., 2025).

This work addresses this gap by integrating soil moisture gradients, distributed directional estimation, and CPG-based locomotion within a single physical modular robotic platform.

## 3 Methodology

This section presents the Soil-Moisture Gradient Distributed Navigation (SMG-DN) control architecture. It is restricted to the methodological formulation of the proposed approach and is organized into three parts. Section 3.1 formulates the navigation problem and defines the exploration objective. Section 3.2 describes the distributed navigation layer responsible for estimating movement direction from soil moisture gradients and obstacle proximity. Section 3.3 describes the CPG-based locomotion layer that transforms the estimated direction into coordinated physical displacement.

### 3.1 Problem formulation

The controller formulates exploration as a navigation problem within a spatially varying soil moisture field. Let $\Omega \subset R^{2}$ denote the exploration domain and let θ(x) represent the soil moisture distribution expressed as volumetric water content, where x∈Ω denotes the robot position.

The objective of the navigation process is to guide the robotic system toward regions with higher soil moisture while avoiding obstacles. The controller estimates movement direction using local measurements and information exchanged among neighboring modules. Under this formulation, the exploration objective is represented through the maximization of the navigation potential defined in Equation 1.

$$\underset{x\left(t\right)}{\max}\int_{0}^{T}\overset{M}{\underset{i=1}{\sum }}\Phi^{\left(i\right)}\left(x\left(t\right),k\right)\;dt\;\text{s.t.}\;x\left(t\right)\in \Omega ,$$ (1)

where $\Phi^{\left(i\right)}\left(x,k\right)$ denotes the local navigation potential of module i , and M is the number of modules considered in the navigation process. The local potential combines attraction toward regions with higher soil moisture and repulsion associated with obstacle proximity.

The sequence through which the navigation process transforms environmental information into movement is summarized in Equation 2.

$$\theta_{s}^{\left(i\right)}\left(k\right)\to {\hat{\theta }}^{\left(i\right)}\left(x,k\right)\to \Phi^{\left(i\right)}\left(x,k\right)\to u_{i}\left(k\right)\to \dot{x}\left(t\right),$$ (2)

where $\theta_{s}^{\left(i\right)}\left(k\right)$ denotes the soil moisture measurement of sensor s in module i , ${\hat{\theta }}^{\left(i\right)}\left(x,k\right)$ is the local soil moisture estimate, $\Phi^{\left(i\right)}\left(x,k\right)$ is the local navigation potential, $u_{i}\left(k\right)$ denotes the cooperative movement direction estimated for module i , and $\dot{x}\left(t\right)$ represents the robot motion.

The architecture separates navigation from locomotion: the navigation layer estimates a movement direction from environmental information, while the locomotion layer transforms this directional estimate into actuator commands generated by coupled oscillators. The robotic system operates under three constraints: environmental measurements are restricted by the sensing range of the installed sensors, information exchange is limited to local communication among neighboring modules, and obstacle interactions modify the available movement directions and influence the navigation process.

### 3.2 Distributed navigation layer

The navigation layer estimates movement direction from local soil moisture measurements and obstacle perception. Environmental information is acquired through sensing modules distributed along the robotic structure and combined through local communication among neighboring modules.

The local soil moisture estimate is computed using Equation 3.

$${\hat{\theta }}^{\left(i\right)}\left(x,k\right)=\frac{\sum_{s=1}^{N_{s}^{\left(i\right)}}\gamma_{s}\theta_{s}^{\left(i\right)}\left(k\right)}{\sum_{s=1}^{N_{s}^{\left(i\right)}}\gamma_{s}},$$ (3)

where $\theta_{s}^{\left(i\right)}\left(k\right)$ denotes the measurement provided by sensor s of module i at control cycle k , $N_{s}^{\left(i\right)}$ is the number of active moisture sensors associated with module i , and $\gamma_{s}$ is the weighting coefficient associated with each measurement. In the experiments, $\gamma_{s}=1$ was used for all active moisture sensors.

The estimated moisture value defines the navigation potential according to Equation 4.

$$\Phi^{\left(i\right)}\left(x,k\right)=\psi \left({\hat{\theta }}^{\left(i\right)}\left(x,k\right)\right)-\lambda^{\left(i\right)}\left(x,k\right),$$ (4)

where ψ(⋅) represents the attraction term associated with soil moisture and $\lambda^{\left(i\right)}\left(x,k\right)$ represents the obstacle avoidance term of module i derived from proximity measurements.

The moisture utility is modeled using the logistic function presented in Equation 5.

$$\psi \left(\theta \right)=\frac{1}{1+e^{-\alpha \left(\theta -\theta_{0}\right)}},$$ (5)

where α controls the slope of the response and $\theta_{0}$ defines the reference moisture level around which the transition occurs. Since the soil moisture measurements were normalized to the interval (0, 1), the parameters were set to α=10 and $\theta_{0}=0.5$ .

The obstacle avoidance term is defined as

$$\lambda^{\left(i\right)}\left(x,k\right)=\kappa_{o}\underset{f\in F}{\max}{\hat{p}}_{f}^{\left(i\right)}\left(k\right),$$ (6)

where $\kappa_{o}$ is the obstacle penalty coefficient, F is the finite set of candidate movement directions, and ${\hat{p}}_{f}^{\left(i\right)}\left(k\right)$ is the normalized obstacle proximity associated with module i and candidate direction f . Since the moisture and proximity terms were normalized to the interval (0, 1), the obstacle penalty coefficient was set to $\kappa_{o}=1$ .

The normalized obstacle proximity is computed as

$${\hat{p}}_{f}^{\left(i\right)}\left(k\right)=\underset{s\in S_{f}^{\left(i\right)}}{\max}\left[{sat}_{\left[0,1\right]}\left(\frac{d_{\max}-d_{s}^{\left(i\right)}\left(k\right)}{d_{\max}-d_{\min}}\right)\right],$$ (7)

where $d_{s}^{\left(i\right)}\left(k\right)$ is the distance estimated by proximity sensor s of module i , $S_{f}^{\left(i\right)}$ is the set of sensors associated with candidate direction f , and $d_{\min}$ and $d_{\max}$ define the minimum and maximum sensing distances considered for obstacle avoidance. The values $d_{\min}=20\;mm$ and $d_{\max}=200\;mm$ were used. The operator ${sat}_{\left[0,1\right]}\left(\cdot \right)$ limits the result to the interval [0, 1]. Therefore, ${\hat{p}}_{f}^{\left(i\right)}\left(k\right)=0$ indicates no obstacle influence in direction f , whereas ${\hat{p}}_{f}^{\left(i\right)}\left(k\right)=1$ indicates maximum obstacle proximity. This quantity is local, normalized, distance-based, and computed from the subset of proximity sensors associated with each candidate direction; therefore, it does not require a global obstacle map or accumulated external information.

The position of a modular chain is approximated through the barycentric representation defined for each modular subset in Equation 8.

$$x_{\text{bar}}^{\left(j\right)}\left(t\right)=\frac{1}{M_{j}}\overset{M_{j}}{\underset{i=1}{\sum }}x_{i}^{\left(j\right)}\left(t\right),$$ (8)

where $x_{\text{bar}}^{\left(j\right)}\left(t\right)$ is the barycentric position of subset j , $M_{j}$ is the number of modules in that subset, and $x_{i}^{\left(j\right)}\left(t\right)$ is the position of module i in subset j . For multiple modular subsets, the global barycentric position is defined by Equation 9.

$$x_{\text{bar}}\left(t\right)=\frac{1}{\sum_{j=1}^{K}M_{j}}\overset{K}{\underset{j=1}{\sum }}\overset{M_{j}}{\underset{i=1}{\sum }}x_{i}^{\left(j\right)}\left(t\right),$$ (9)

where K is the number of modular subsets. The desired movement direction corresponds to the direction of maximum increase of the navigation potential,

$$u^{⋆}\left(t\right)=\frac{\nabla \Phi^{\left(i\right)}\left(x_{\text{bar}}\left(t\right),k\right)}{\left\|\nabla \Phi^{\left(i\right)}\left(x_{\text{bar}}\left(t\right),k\right)\right\|+\varepsilon },$$ (10)

where ε is a positive constant used to avoid division by zero; in this implementation, $\varepsilon =10^{-6}$ was used.

The direction defined by Equation 10 represents a continuous formulation of the navigation objective. Since the modular robot does not have access to the complete environmental field, this direction is approximated through a finite set of candidate headings evaluated from local measurements. The corresponding kinematic model is presented in Equation 11.

$$\dot{x}\left(t\right)=v\left(t\right)u^{⋆}\left(t\right),$$ (11)

where v(t) is the translational velocity magnitude.

Each module evaluates a finite set of candidate directions f∈F using a directional utility function,

$$J_{f}^{\left(i\right)}\left(k\right)=\alpha_{J}\Delta {\hat{\theta }}^{\left(i\right)}\left(f,k\right)-\beta_{J}{\hat{p}}_{f}^{\left(i\right)}\left(k\right),$$ (12)

where $\Delta {\hat{\theta }}^{\left(i\right)}\left(f,k\right)$ represents the estimated soil moisture variation in direction f , and ${\hat{p}}_{f}^{\left(i\right)}\left(k\right)$ represents normalized obstacle proximity. The coefficients $\alpha_{J}$ and $\beta_{J}$ regulate the contribution of the moisture variation and obstacle proximity terms, respectively. Since both terms were normalized to the interval [0, 1], the values $\alpha_{J}=1$ and $\beta_{J}=1$ were used.

The estimated soil moisture variation is defined by Equation 13.

$$\Delta {\hat{\theta }}^{\left(i\right)}\left(f,k\right)={\hat{\theta }}_{f}^{\left(i\right)}\left(k\right)-{\hat{\theta }}^{\left(i\right)}\left(k\right),$$ (13)

where ${\hat{\theta }}^{\left(i\right)}\left(k\right)$ is the local moisture estimate of module i , and ${\hat{\theta }}_{f}^{\left(i\right)}\left(k\right)$ is the directional moisture estimate associated with candidate direction f . This value is computed from the local moisture measurements associated with the module or neighboring sensing locations aligned with candidate direction f .

The preferred direction is selected according to Equation 14.

$$f_{i}^{⋆}\left(k\right)=\arg\underset{f\in F}{\max}J_{f}^{\left(i\right)}\left(k\right).$$ (14)

Neighboring modules exchange directional information and compute a cooperative movement vector,

$$u_{i}\left(k\right)=\frac{\sum_{j\in N\left(i\right)}w_{ij}e_{f_{j}^{⋆}\left(k\right)}}{\sum_{j\in N\left(i\right)}w_{ij}},$$ (15)

where N(i) denotes the set of neighboring modules, $w_{ij}$ is the weighting coefficient associated with information received from module j , and $e_{f_{j}^{⋆}\left(k\right)}$ is the unit vector associated with the selected direction of module j . In the experiments, $w_{ij}=1$ was used for neighboring modules.

The discrete position update is given by Equation 16.

$$x_{i}\left(k+1\right)=x_{i}\left(k\right)+vT_{s}u_{i}\left(k\right),$$ (16)

where $T_{s}$ is the sampling period and v is the nominal translational velocity used during the discrete position update. In this implementation, $T_{s}=50\;ms$ and v=0.10 m/s were used, corresponding to a nominal displacement of 5 mm per control cycle.

### 3.3 CPG-based locomotion layer

The locomotion layer transforms the cooperative direction estimated by the navigation layer into physical motion. The locomotion mechanism employs a network of coupled Central Pattern Generators, where each module is associated with an oscillator that generates actuator commands.

The oscillator dynamics and the corresponding actuator command are defined by Equations 17, 18, respectively.

$${\dot{\varphi }}_{m}=\omega_{m}+\underset{\ell \in N\left(m\right)}{\sum }K_{m\ell }\sin\left(\varphi_{\ell }-\varphi_{m}-\Delta_{m\ell }\left(k\right)\right),$$ (17)

and the corresponding actuator command is

$$y_{m}=A_{m}\sin\left(\varphi_{m}\right),$$ (18)

where $\varphi_{m}$ denotes the oscillator phase, $\omega_{m}$ is the natural frequency, $A_{m}$ is the oscillation amplitude, and $y_{m}$ is the actuator command applied to module m . In this implementation, a common oscillator frequency $\omega_{m}=2\pi \;rad/s$ and an oscillation amplitude $A_{m}=0.35\;rad$ were used for all modules.

The coupling coefficient is defined by Equation 19.

$$K_{m\ell }=K_{0}\eta_{m\ell },$$ (19)

where $K_{0}$ is the coupling constant and $\eta_{m\ell }$ is the topological coupling coefficient between modules m and ℓ . In this implementation, $K_{0}=1.5$ was used. The value $\eta_{m\ell }=1$ was used for directly connected neighboring modules, and $\eta_{m\ell }=0$ was used otherwise.

The nominal phase lag between oscillators is defined by Equation 20.

$$\Delta_{m\ell }^{0}=d_{0}d\left(m,\ell \right),$$ (20)

where $d_{0}$ is the nominal phase lag and d(m,ℓ) denotes the topological distance between modules. In this implementation, $d_{0}=\pi /4\;rad$ was used as the nominal phase lag between adjacent modules.

The navigation layer modifies the oscillator coordination according to Equation 21.

$$\Delta_{m\ell }\left(k\right)=\Delta_{m\ell }^{0}+\delta_{m\ell }\left(k\right),$$ (21)

where $\delta_{m\ell }\left(k\right)$ is the phase correction term generated from the movement direction estimated by the navigation layer. The phase correction term is defined by Equation 22.

$$\delta_{m\ell }\left(k\right)=\kappa_{\delta }s_{m\ell }\Delta \phi_{u}\left(k\right),$$ (22)

where $\kappa_{\delta }$ is the phase correction gain, $s_{m\ell }$ defines the sign of the correction according to the relative position of modules m and ℓ , and $\Delta \phi_{u}\left(k\right)$ is the angular difference between the current locomotion direction and the direction estimated by the navigation layer. The sign term was defined as $s_{m\ell }=1$ when the phase correction advances the traveling wave in the selected movement direction and $s_{m\ell }=-1$ when it delays the traveling wave. The value $\kappa_{\delta }=1$ was used after normalizing $\Delta \phi_{u}\left(k\right)$ .

The locomotion layer updates the oscillator states, exchanges synchronization information among neighboring modules, and generates actuator commands at each control cycle. The resulting phase relationships produce traveling waves along the modular chain and generate physical displacement. The same locomotion layer was used for all navigation strategies during the experimental comparison; therefore, the differences among methods are associated with the directional estimation process.

The control, sensing, navigation, and locomotion parameters used in the experimental implementation are summarized in Table 2.

**TABLE 2:** Control, sensing, navigation, and locomotion parameters used in the experimental implementation.

Parameter	Value	Description
$T_{s}$	50 ms	Controller sampling period
$T_{\text{prox}}$	20 ms	Proximity sensing update period associated with the VCNL4010 sensors
$T_{\text{adc}}$	100 ms	Infrared sensing update period used for soil moisture estimation
$T_{\text{dxl}}$	20 ms	Actuator command update period for the dynamixel AX-12 A servomotors
$T_{\text{esp}}$	50 ms	Wireless communication update period
$T_{\text{udp}}$	50 ms	UDP telemetry transmission period
$\gamma_{s}$	1	Soil moisture sensor weight
α	10	Slope parameter of the logistic moisture utility
$\theta_{0}$	0.5	References moisture level of the logistic moisture utility
$\kappa_{o}$	1	Obstacle penalty coefficient
$\alpha_{J}$	1	Soil moisture variation weight in the directional utility function
$\beta_{J}$	1	Obstacle proximity weight in the directional utility function
$d_{\min}$	20 mm	Minimum distance for maximum obstacle influence
$d_{\max}$	200 mm	Maximum distance considered for obstacle avoidance
ε	$10^{-6}$	Constant used in the normalized gradient direction
v	0.10 m/s	Nominal translational velocity used in the discrete position update
$\omega_{m}$	2π rad/s	Natural frequency of oscillator m
$A_{m}$	0.35 rad	Oscillation amplitude of module m
$K_{0}$	1.5	Coupling constant between neighboring oscillators
$d_{0}$	π/4 rad	Nominal phase lag between adjacent modules
$\kappa_{\delta }$	1	Phase correction gain
$\eta_{m\ell }$	1/0	Topological coupling coefficient; 1 for connected modules and 0 otherwise
$s_{m\ell }$	{−1,1}	Sign of the phase correction according to the relative module position

## 4 Experimental setup

This section describes the experimental setup used to validate the proposed control architecture. It includes the robotic platform, the reference algorithms used for comparison, and the experimental protocol. This organization separates the proposed method, presented in Section 3, from the hardware implementation and evaluation procedure. The reference algorithms are included only as baseline methods and are not components of the proposed control architecture.

### 4.1 Robotic platform

**FIGURE 1:**
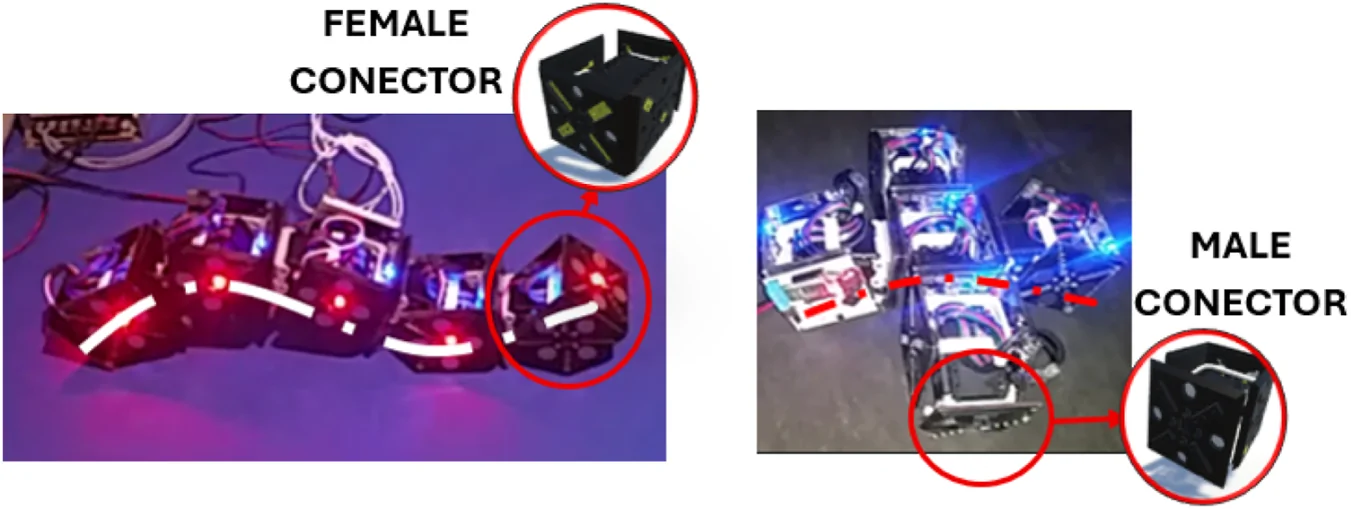
EMeRGe modular robot configured as a chain-type structure.

The experimental validation employs a modified version of the EMeRGe modular robot (*Easy Modular Embodied Robot Generator*, Figure 1. The robotic platform consists of a set of semi-reconfigurable modules interconnected through magnetic coupling mechanisms. Each module provides one rotational degree of freedom actuated by a Dynamixel AX-12A servomotor.

Each module integrates sensing, wireless communication, local processing, and actuation capabilities. The mechanical structure contains four functional faces, including three female connectors and one male connector, with neodymium magnets that enable mechanical coupling among neighboring modules.

The proposed navigation strategy is implemented on the EMeRGe platform to evaluate distributed directional estimation under physical operating conditions. The formulation is not restricted to a single chain geometry because the navigation layer uses local environmental measurements and neighbor-to-neighbor communication. However, the experimental validation reported in this work is limited to the EMeRGe modular platform and the tested configurations.

Each module integrates an ESP32-WROOM-32E microcontroller for wireless communication, telemetry transmission, and local coordination tasks. The low-level actuation commands are transmitted to the Dynamixel AX-12A servomotors through a UART-TTL interface. This specification is included to define the computational platform used during the experiments and to support the analysis of real-time execution within the 50 ms control cycle.

The sensing subsystem acquires the environmental information required by the navigation layer. Proximity measurements are obtained through VCNL4010 sensors mounted on the module faces, whereas soil moisture measurements are obtained through an auxiliary sensing module based on the AS7341 spectral sensor operating in the infrared range. The sensing module estimates soil moisture from reflected infrared radiation and converts the sensor response into volumetric water content through the calibration procedure described in Section 4.3. This configuration enables the robotic system to estimate local soil moisture conditions while detecting nearby obstacles.

**FIGURE 2:**
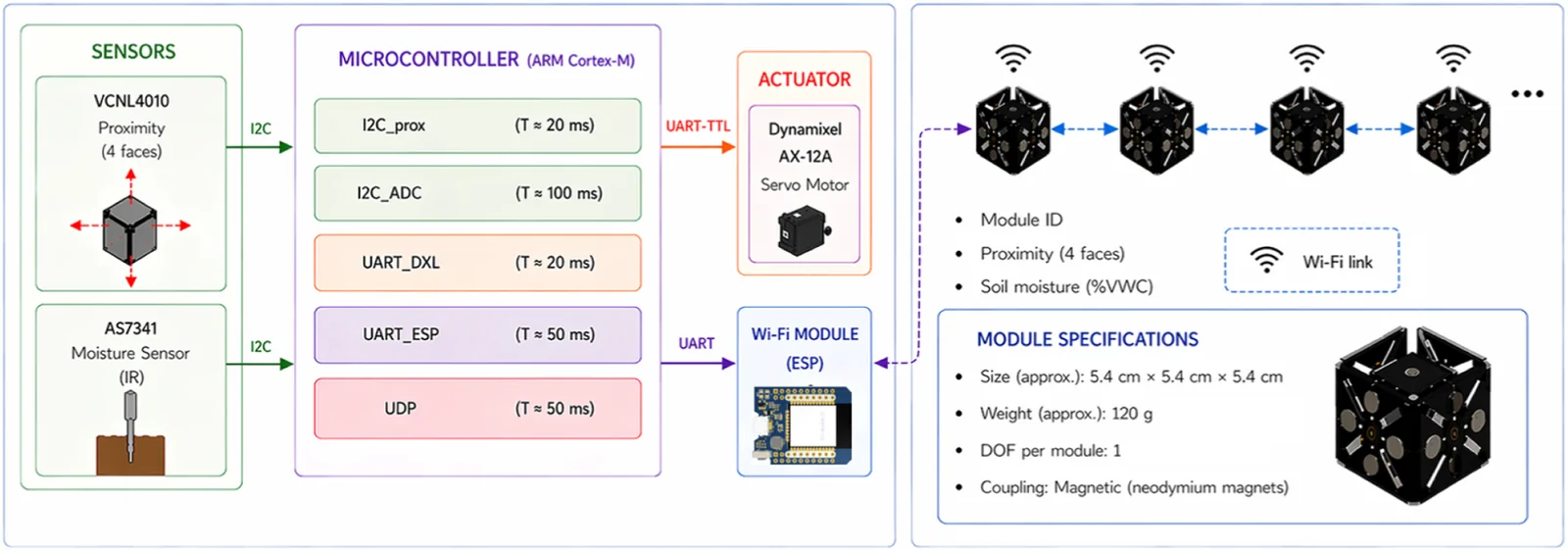
Distributed sensing, communication, and control architecture implemented on the modular robotic platform.

The communication subsystem exchanges navigation information among neighboring modules through wireless links, including module identifiers, soil moisture measurements, obstacle perception data, and synchronization variables required by the distributed control architecture. The actuation subsystem employs Dynamixel AX-12A servomotors connected through a UART-TTL interface. The locomotion layer generates coordinated commands from the oscillator states of the CPG network and transmits these commands to the actuators to produce physical motion. Figure 2 summarizes the sensing, communication, processing, and actuation components integrated within the robotic platform.

### 4.2 Reference algorithms

The reference algorithms are not part of the proposed control architecture. They are included only as baseline methods to compare directional estimation performance under the same sensing, locomotion, and experimental conditions. In all cases, the estimated movement direction is transformed into physical displacement through the same CPG-based locomotion layer.

All evaluated methods use the same directional utility function defined in Equation 12. Therefore, the comparison is based on the mechanism used to estimate or select the movement direction, rather than on differences in sensing, obstacle representation, utility definition, or locomotion. The coefficients $\alpha_{J}$ and $\beta_{J}$ were kept equal to the values used by the proposed controller, namely, $\alpha_{J}=1$ and $\beta_{J}=1$ .

The comparative evaluation considers three baseline methods selected to represent different directional estimation mechanisms under the same sensing, locomotion, and experimental conditions. Ant Colony Optimization and Genetic Algorithm were included as iterative optimization-based baselines because both methods are commonly used for discrete search and path-selection problems. However, since the proposed SMG-DN controller follows local variations of the navigation potential, a gradient-based Hill Climbing baseline was also included as a directly comparable method. This additional baseline allows the evaluation to distinguish the effect of the proposed distributed gradient formulation from the effect of using a local gradient-following strategy in general. All baseline methods operate on the same set of candidate directions F and employ the same environmental information available to the proposed controller.

#### 4.2.1 Ant colony optimization

The Ant Colony Optimization algorithm estimates movement direction through a probabilistic decision process that combines accumulated information and local heuristic evaluation. Each candidate direction is evaluated according to the directional utility defined in Equation 12 and assigned a selection probability,

$$P_{f}\left(k\right)=\frac{\tau_{f}{\left(k\right)}^{\gamma }\eta_{f}{\left(k\right)}^{\rho }}{\sum_{q\in F}\tau_{q}{\left(k\right)}^{\gamma }\eta_{q}{\left(k\right)}^{\rho }},$$ (23)

where $\tau_{f}\left(k\right)$ denotes the accumulated information associated with direction f , $\eta_{f}\left(k\right)$ represents the non-negative heuristic value derived from the directional utility, and γ and ρ regulate the influence of accumulated information and heuristic evaluation, respectively. The heuristic value was computed as

$$\eta_{f}\left(k\right)=J_{f}^{\left(i\right)}\left(k\right)-\underset{q\in F}{\min}J_{q}^{\left(i\right)}\left(k\right)+\epsilon_{\eta },$$ (24)

where $\epsilon_{\eta }=10^{-6}$ was used to keep the heuristic value strictly positive.

The accumulated information evolves according to

$$\tau_{f}\left(k+1\right)=\left(1-\lambda_{\tau }\right)\tau_{f}\left(k\right)+\Delta \tau_{f}\left(k\right),$$ (25)

where $\lambda_{\tau }$ is the evaporation factor and $\Delta \tau_{f}\left(k\right)$ is the reinforcement term associated with the selected direction. The symbol $\lambda_{\tau }$ is used to avoid confusion with the obstacle avoidance term $\lambda^{\left(i\right)}\left(x,k\right)$ defined in Equation 6. The reinforcement term was computed according to Equation 26.

$$\Delta \tau_{f}\left(k\right)=\left\{\begin{array}{cc} \eta_{f}\left(k\right), & f=f_{i}^{⋆}\left(k\right),\\ 0, & f\neq f_{i}^{⋆}\left(k\right), \end{array}\right.$$ (26)

where $\eta_{f}\left(k\right)$ is the non-negative heuristic value defined in Equation 24.

Algorithm 1 summarizes the implementation adopted in this work.

Algorithm 1 Directional estimation using ACO.

**Require:** F , $\theta_{s}^{\left(i\right)}\left(k\right)$ , ${\hat{p}}_{f}^{\left(i\right)}\left(k\right)$ , $\tau_{f}\left(k\right)$

**Ensure:** $f_{i}^{⋆}\left(k\right)$

  1: **for** each f∈F **do**
  2:   Compute $J_{f}^{\left(i\right)}\left(k\right)$

  3: **end for**
  4: **for** each f∈F **do**
  5:   Compute $\eta_{f}\left(k\right)$ using Equation 24
  6: end for
  7: Compute $P_{f}\left(k\right)$ using Equation 23
  8: Select $f_{i}^{⋆}\left(k\right)$ according to $P_{f}\left(k\right)$

  9: Update $\tau_{f}\left(k+1\right)$ using Equation 25
  10 Send $f_{i}^{⋆}\left(k\right)$ to the locomotion layer

#### 4.2.2 Genetic algorithm

The Genetic Algorithm estimates movement direction through an evolutionary process in which each individual represents a candidate direction in F . The Genetic Algorithm optimization problem is defined by Equation 27.

$$f_{i}^{⋆}\left(k\right)=\arg\underset{d_{r}\in P\left(k\right)}{\max}J\left(d_{r}\right),$$ (27)

where $J\left(d_{r}\right)$ corresponds to the directional utility $J_{f}^{\left(i\right)}\left(k\right)$ defined in Equation 12, evaluated for the candidate direction represented by individual $d_{r}$ .

The population evolves according to Equation 28.

$$P\left(k+1\right)=S\left(C\left(M\left(P\left(k\right)\right)\right)\right),$$ (28)

where S , C , and M denote the selection, recombination, and mutation operators, respectively. In this implementation, selection retained the highest-utility individuals, recombination exchanged candidate directions between selected individuals, and mutation replaced a candidate direction with another direction randomly sampled from F with probability $p_{m}$ .

Algorithm 2 summarizes the implementation adopted in this work.

Algorithm 2 Directional estimation using GA.

**Require:** F , $\theta_{s}^{\left(i\right)}\left(k\right)$ , ${\hat{p}}_{f}^{\left(i\right)}\left(k\right)$

**Ensure:** $f_{i}^{⋆}\left(k\right)$

 1: Initialize population P(k)

 2: **for** each $d_{r}\in P\left(k\right)$ **do**
 3:   Compute $J\left(d_{r}\right)$ using Equation 12
 4: **end for**
 5: Apply selection operator S

 6: Apply recombination operator C

 7: Apply mutation operator M

 8: Select $f_{i}^{⋆}\left(k\right)=\arg\max J\left(d_{r}\right)$

 9: Send $f_{i}^{⋆}\left(k\right)$ to the locomotion layer

#### 4.2.3 Hill climbing

The Hill Climbing baseline estimates the movement direction by selecting the candidate direction with the highest local directional utility. This method was included as a gradient-based reference because it follows local increases of the same navigation utility used by the proposed controller, but without the distributed cooperative aggregation among neighboring modules.

The direction selected by the Hill Climbing method is defined by Equation 29.

$$f_{i}^{⋆}\left(k\right)=\arg\underset{f\in F}{\max}J_{f}^{\left(i\right)}\left(k\right),$$ (29)

where $J_{f}^{\left(i\right)}\left(k\right)$ is the directional utility defined in Equation 12. Unlike the proposed SMG-DN controller, the Hill Climbing baseline does not combine directional decisions from neighboring modules through the cooperative movement vector in Equation 15. Therefore, this baseline evaluates the contribution of distributed directional cooperation separately from local gradient-following behavior.

Algorithm 3 summarizes the Hill Climbing directional estimation procedure.

Algorithm 3 Directional estimation using Hill Climbing.

**Require:** F , $\theta_{s}^{\left(i\right)}\left(k\right)$ , ${\hat{p}}_{f}^{\left(i\right)}\left(k\right)$

**Ensure:** $f_{i}^{⋆}\left(k\right)$

 1: **for** each f∈F **do**
 2:   Compute $J_{f}^{\left(i\right)}\left(k\right)$ using Equation 12
 3: **end for**
 4: Select $f_{i}^{⋆}\left(k\right)=\arg\max_{f\in F}J_{f}^{\left(i\right)}\left(k\right)$

 5: Send $f_{i}^{⋆}\left(k\right)$ to the locomotion layer

The parameters of the reference algorithms were selected to ensure real-time execution within the control loop. The ACO parameters were set to γ=1 , ρ=2 , $\lambda_{\tau }=0.10$ , and $\tau_{f}\left(0\right)=1$ for all candidate directions. The GA used a population size of $N_{p}=12$ , a mutation rate of $p_{m}=0.10$ , and G=5 generations per control cycle. The Hill Climbing baseline did not require population, pheromone, or mutation parameters because this method directly selects the candidate direction with the maximum local utility. These values were selected to keep the computational cost compatible with the controller sampling period $T_{s}=50\;ms$ . Table 5 presents the computation time obtained for each directional estimation method within the 50 ms control cycle.

All baseline methods supply the selected direction $f_{i}^{⋆}\left(k\right)$ to the same CPG-based locomotion layer; therefore, the comparison isolates the directional estimation strategy.

### 4.3 Experimental protocol

**FIGURE 3:**
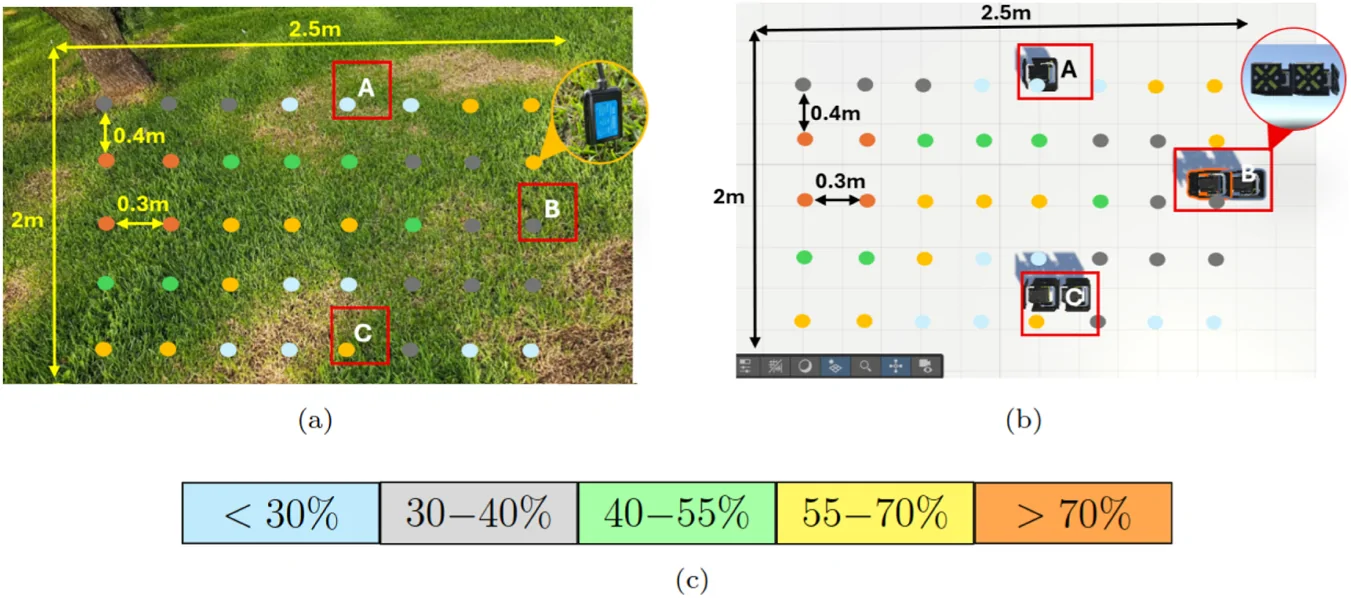
Experimental field used for soil moisture characterization and sensor calibration. **(a)** Physical environment **(b)** Virtual environment **(c)** Relative humidity scale.

The experimental validation was conducted in two physical stages. The first stage consisted of characterizing the soil moisture distribution within a test area of approximately 2 m×2.5 m using a commercial moisture meter with an effective penetration depth of approximately 50 mm . These measurements were used as reference values for calibrating the sensing subsystem integrated into the robotic platform. Figure 3 illustrates the experimental field and the spatial distribution of the acquired measurements.

The sensing subsystem employs an auxiliary infrared module based on the AS7341 spectral sensor. The infrared signal acquired by the sensor is associated with soil surface reflectance and therefore does not directly correspond to a moisture measurement. A calibration procedure was performed using paired measurements of reference soil moisture and infrared reflectance. The relationship between the infrared signal and reference soil moisture was modeled through the linear regression presented in Equation 30.

$$\theta_{i}=a_{c}s_{i}+b_{c},$$ (30)

where $s_{i}$ denotes the infrared signal, $\theta_{i}$ is the estimated volumetric water content, and $a_{c}$ and $b_{c}$ are calibration coefficients.

Table 3 summarizes representative calibration measurements used to estimate the regression model.

**TABLE 3:** Relationship between reference moisture measurements, infrared sensor response, and estimated soil moisture values.

Point	References moisture (VWC)	Ir signal $\left(s_{i}\right)$	Estimated moisture (VWC)
1	22	120	23
2	24	135	25
3	27	160	28
4	29	180	30
5	32	210	33
6	35	240	36
7	38	270	39
8	42	300	43

The calibrated measurements define discrete samples of the scalar field according to Equation 31.

$$\theta \left(x_{i}\right)\approx \theta_{i},\;i=1,\ldots ,N,$$ (31)

where $\theta_{i}$ denotes the calibrated moisture value at sampling position $x_{i}$ . These calibrated measurements represent discrete samples of the spatial soil moisture distribution within the exploration domain. This field is not globally available to the robot and is estimated locally during operation through distributed sensing and communication.

The second stage consisted of evaluating the navigation strategy under heterogeneous environmental conditions. The experimental domain was discretized into grids, where each cell represents a region with a characteristic moisture level. Three initial positions, denoted as A, B, and C, were selected to represent different local gradient conditions. Additional experiments were conducted using controlled surfaces with different reflectance properties and obstacle configurations in order to evaluate navigation and obstacle avoidance performance.

**FIGURE 4:**
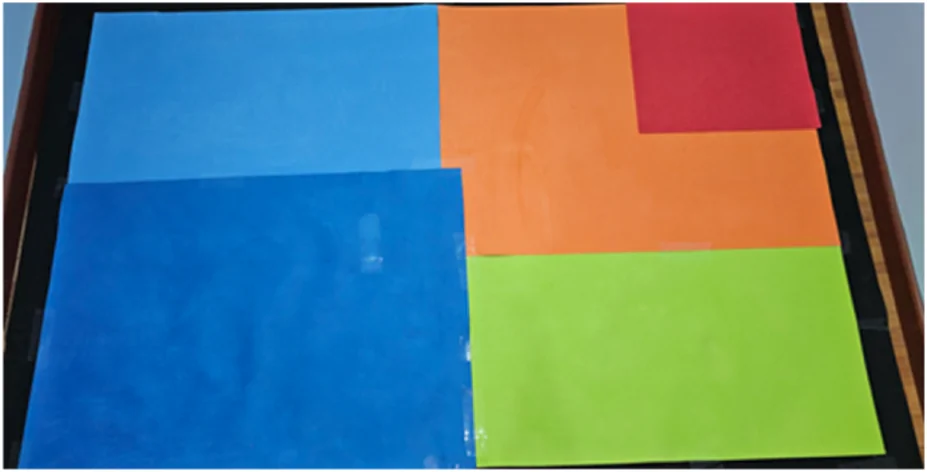
Controlled experimental surface.

**FIGURE 5:**
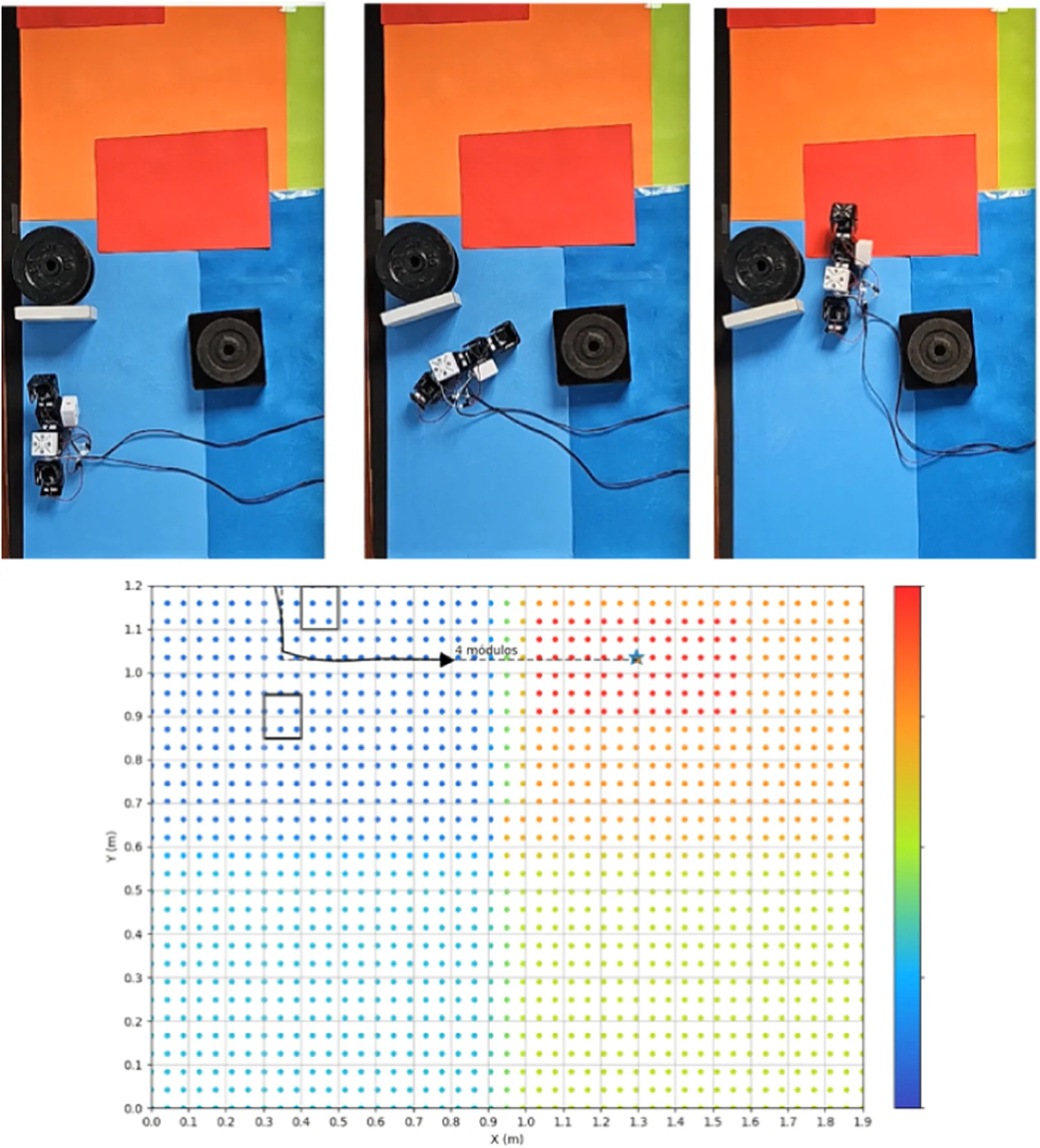
Representative navigation sequence.

**FIGURE 6:**
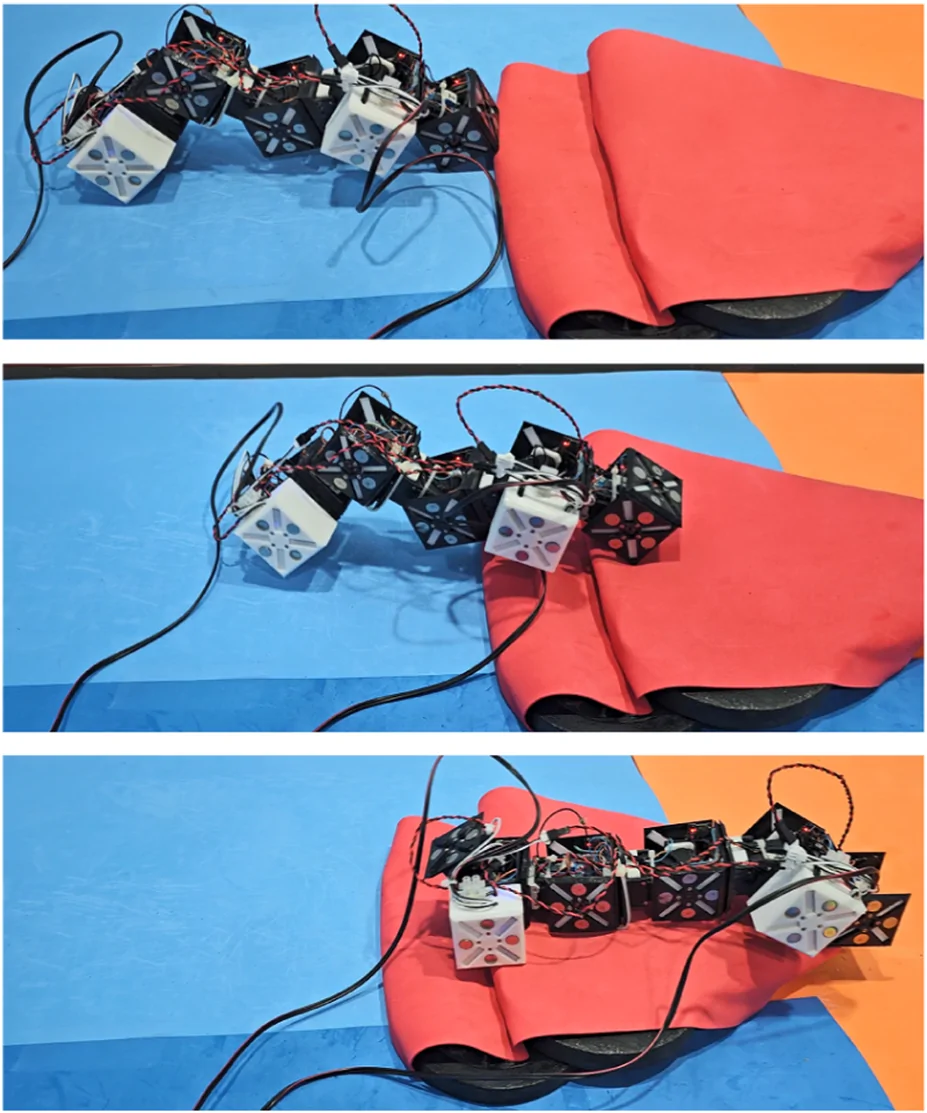
Stepped obstacle configuration.

Figures 4–6 correspond to physical experimental conditions used during validation and do not represent a simulation environment. These figures show the controlled experimental surface, a representative navigation sequence, and the obstacle configuration used to evaluate proximity-based directional selection.

The experimental protocol considered three modular configurations in order to evaluate whether the navigation strategy remained applicable under different spatial distributions of sensing points and locomotion constraints. Table 4 summarizes the configurations used during validation and the role of each configuration in the experimental design.

**TABLE 4:** Robot configurations evaluated during the experimental validation.

Configuration	Number of modules	Purpose in the evaluation
Three-module chain configuration	3 modules	Baseline modular arrangement used to evaluate gradient-based navigation and CPG locomotion under compact sensing distribution
Five-module chain configuration	5 modules	Configuration used to evaluate the effect of a larger spatial distribution of sensing points and longer traveling waves
Cross-type modular configuration	5 modules	Configuration used to evaluate navigation performance when the modular structure introduces lateral sensing positions and additional coordination requirements

**TABLE 5:** Computation time of the directional estimation methods within the 50 ms control cycle.

Method	Mean time (ms)	Std. dev. (ms)	Maximum time (ms)
SMG-DN	3.84	0.42	4.91
Hill climbing	2.31	0.28	3.07
ACO	8.76	1.14	11.92
GA	14.63	2.08	19.74

Using the configurations summarized in Table 4, each combination of initial condition, robot configuration, and controller was executed ten times. The evaluation considered the proposed SMG-DN controller, Hill Climbing, Ant Colony Optimization, and Genetic Algorithm, while maintaining identical sensing and locomotion conditions. This configuration resulted in 360 physical trials, corresponding to three initial conditions, three robot configurations, four controllers, and ten repetitions per combination.

The real-time feasibility of each method was evaluated by measuring the computation time required to estimate the movement direction within each control cycle. The computation time was obtained on the ESP32-WROOM-32 E platform using timer readings before and after the directional estimation routine. The measured value did not include sensor acquisition, wireless transmission, or actuator command transmission. For each method, the mean computation time, standard deviation, and maximum observed computation time were computed over all executions and compared with the controller sampling period $T_{s}=50\;ms$ .

All evaluated methods completed the directional estimation routine below the 50 ms control cycle. The SMG-DN controller required higher computation time than Hill Climbing because the method includes cooperative aggregation among neighboring modules, but the SMG-DN controller required lower computation time than ACO and GA because the method does not rely on pheromone updating or population-based search.

Experimental data were transmitted from the robot modules to an external computer through a Wi-Fi communication link operating at a sampling interval of approximately $T_{s}=50\;ms$ , equivalent to 20 Hz . Each transmitted packet contained the module identifier, infrared signal, estimated moisture, proximity measurements, and locomotion variables associated with the CPG network. Data were received through a UDP communication channel and stored in CSV format for subsequent analysis.

Algorithm 4 summarizes the acquisition and logging procedure adopted during the experiments. Algorithm 5 summarizes the procedure used to measure the computation time of each directional estimation method.

Algorithm 4 Data acquisition and logging.

**Require:** $T_{s}$ , UDP port, $a_{c}$ , $b_{c}$

**Ensure:** Dataset D

 1: Initialize Wi-Fi communication
 2: Configure UDP socket
 3: D←∅

 4: **while** experiment active **do**
 5:   Receive packet $p_{k}$

 6:   Assign timestamp $t_{\text{PC}}$

 7:   Decode $\left(ID,t_{\text{robot}},s_{i}\left(k\right),{\hat{p}}^{\left(i\right)}\left(k\right),\varphi_{i}\left(k\right),A_{i}\left(k\right),checksum\right)$

 8:   Validate packet integrity
 9:   Compute $\theta_{i}\left(k\right)=a_{c}s_{i}\left(k\right)+b_{c}$

 10:   Store data in D

 11: **end while**
 12: Close communication

Algorithm 5 Computation time measurement.

**Require:** Directional estimation method, control cycle k

**Ensure:** Computation time $t_{\text{comp}}\left(k\right)$

 1: Read ESP32 timer value $t_{0}$

 2: Execute directional estimation routine
 3: Read ESP32 timer value $t_{1}$

 4: Compute $t_{\text{comp}}\left(k\right)=t_{1}-t_{0}$

 5: Store $t_{\text{comp}}\left(k\right)$

Table 6 summarizes the structure of the telemetry packet transmitted by each module.

**TABLE 6:** Structure of the telemetry packet transmitted by each module.

Field	Type	Unit	Description
ID	Uint8	–	Module identifier
$t_{\text{robot}}$	Uint32	ms	Local timestamp generated by the module
$t_{\text{PC}}$	Uint32	ms	Reception timestamp assigned by the PC.
$s_{i}$	Uint16	–	Infrared reflectance signal measured by the AS7341 sensor
$\theta_{i}$	Float	VWC	Estimated soil moisture expressed as volumetric water content
${\hat{p}}^{\left(i\right)}$	Float	–	Module-level normalized obstacle proximity measurement
$\varphi_{i}$	Float	Rad	CPG oscillator phase of the module
$A_{i}$	Float	Rad	CPG oscillator amplitude of the module
Checksum	Uint8	–	Packet integrity verification field

The packet contains module-level proximity measurements, from which the directional proximity terms ${\hat{p}}_{f}^{\left(i\right)}\left(k\right)$ are computed according to Equation 7.

## 5 Results

The experimental evaluation considered three initial positions (A, B, and C), three robot configurations, and four navigation controllers: the proposed Soil-Moisture Gradient Distributed Navigation (SMG-DN) controller, Hill Climbing, Ant Colony Optimization (ACO), and Genetic Algorithm (GA). Each experimental condition was repeated ten times, resulting in a total of 360 physical executions. The complete dataset comprised approximately $1.5\times 10^{5}$ temporal observations collected during physical experiments. The sampling period was maintained at approximately $T_{s}=50\;ms$ throughout all experimental trials.

Table 7 presents the average results grouped by initial condition, integrating the effect of the different robot configurations.

**TABLE 7:** Average performance obtained for each initial condition.

Initial point	Controller	$t_{c}$ (s)	${\bar{\theta }}_{e}$	η	$\sigma_{v}^{2}$
A	SMG-DN	17.9	0.21	0.93	0.020
A	Hill climbing	20.6	0.26	0.90	0.025
A	ACO	22.8	0.30	0.88	0.029
A	GA	24.5	0.28	0.86	0.026
B	SMG-DN	18.7	0.24	0.91	0.022
B	Hill climbing	21.4	0.28	0.89	0.027
B	ACO	23.9	0.33	0.87	0.032
B	GA	25.4	0.30	0.85	0.029
C	SMG-DN	19.3	0.25	0.90	0.023
C	Hill climbing	22.1	0.29	0.88	0.028
C	ACO	24.8	0.35	0.86	0.034
C	GA	26.2	0.32	0.84	0.031

The results indicate that initial condition A, associated with stronger moisture gradients, produced lower convergence times and directional estimation errors than conditions B and C. This trend suggests that the magnitude of the local gradient affects the directional information available to the navigation layer.

Table 8 summarizes the overall performance obtained across all experimental executions. The proposed SMG-DN controller achieved the lowest convergence time, directional estimation error, and velocity variance, while obtaining the highest navigation efficiency among the evaluated methods. Relative to Hill Climbing, SMG-DN reduced convergence time by approximately 13%, indicating that distributed directional cooperation improves local gradient-following behavior. Relative to ACO and GA, the convergence time reductions were approximately 22% and 27%, respectively. In addition to the controller-level comparison, the results were grouped according to the modular configuration used during validation, since the navigation layer estimates the desired direction from distributed environmental measurements, whereas the locomotion layer must execute that direction through a specific morphology. Therefore, differences in sensing distribution, body length, and oscillator coordination can influence the final navigation performance.

**TABLE 8:** Overall performance considering all experimental executions.

Controller	$t_{c}$ (s)	${\bar{\theta }}_{e}$	η	$\sigma_{v}^{2}$
SMG-DN	18.6±1.4	0.23±0.02	0.91±0.01	0.022±0.002
Hill climbing	21.4±1.5	0.28±0.02	0.89±0.01	0.027±0.002
ACO	23.8±1.8	0.33±0.03	0.87±0.01	0.032±0.002
GA	25.4±1.7	0.30±0.02	0.85±0.01	0.029±0.002

**TABLE 9:** Average performance grouped by robot configuration.

Robot configuration	$t_{c}$ (s)	${\bar{\theta }}_{e}$	η	$\sigma_{v}^{2}$
Three-module chain	23.2±3.1	0.30±0.05	0.87±0.03	0.030±0.006
Five-module chain	21.4±2.8	0.26±0.04	0.90±0.02	0.025±0.005
Cross-type modular configuration	22.3±3.0	0.28±0.05	0.88±0.03	0.028±0.005

**TABLE 10:** One-way ANOVA results obtained from the comparison of the evaluated controllers considering all experimental executions.

Metric	F-statistic	p-value	Result
$t_{c}$	24.6	<0.001	Significant
${\bar{\theta }}_{e}$	18.9	<0.001	Significant
η	16.2	<0.001	Significant
$\sigma_{v}^{2}$	12.4	0.001	Significant

**TABLE 11:** Pearson correlation coefficients between the evaluated performance metrics.

Metric pair	Pearson coefficient (r)	Interpretation
$t_{c}$ vs. η	−0.74	Strong inverse relationship
${\bar{\theta }}_{e}$ vs. $\sigma_{v}^{2}$	0.68	Moderate direct relationship
$t_{c}$ vs. $\sigma_{v}^{2}$	0.61	Moderate direct relationship

Table 9 summarizes the average performance grouped by robot configuration. The configuration-level analysis indicates that robot morphology influenced the physical execution of the direction estimated by the navigation layer. The five-module chain achieved the best overall performance, with lower convergence time, lower directional estimation error, higher navigation efficiency, and lower velocity variance, which is consistent with its larger sensing distribution and smoother CPG traveling-wave propagation. In contrast, the three-module chain showed higher convergence time and velocity variance due to its reduced spatial sampling, while the cross-type configuration introduced additional coordination requirements during direction changes. To evaluate whether the observed performance differences among controllers were statistically significant, a one-way ANOVA was performed considering all experimental executions and the four evaluated performance metrics.

Table 10 presents the one-way ANOVA results obtained for the four evaluated performance metrics. The ANOVA results indicate statistically significant differences among the evaluated controllers for all performance metrics. The corresponding p-values remain below the significance threshold, supporting the existence of systematic differences in navigation performance. These differences are consistent with the performance trends reported in Tables 7, 8.

Table 11 presents the Pearson correlation coefficients obtained between the evaluated performance metrics.

The strongest relationship was observed between convergence time and navigation efficiency (r=−0.74) , indicating that faster convergence is associated with more efficient navigation. Positive correlations between directional estimation error and velocity variance indicate that less accurate directional estimates tend to increase motion variability.

**FIGURE 7:**
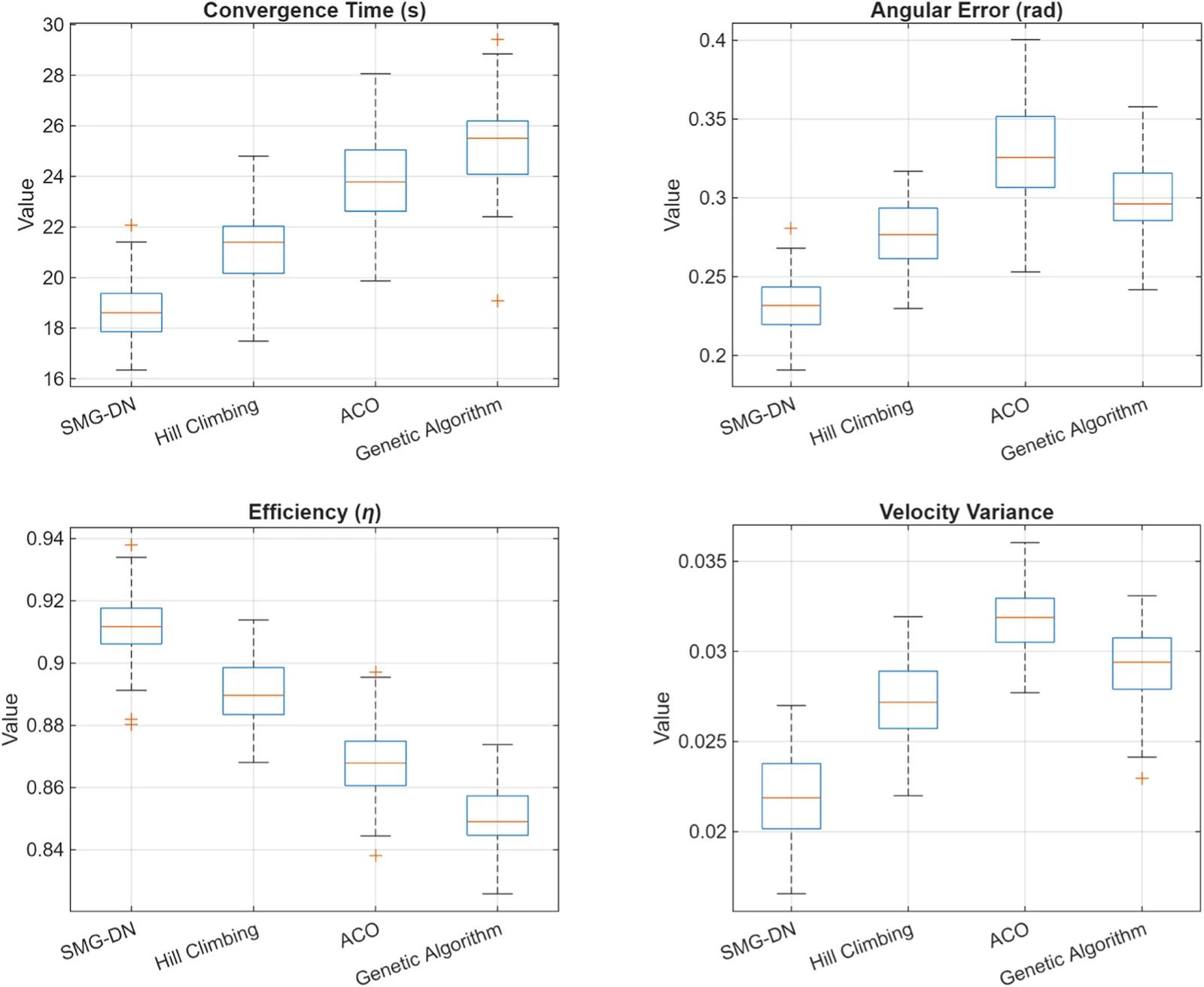
Distribution of the evaluated performance metrics for the considered controllers. The dispersion analysis indicates that SMG-DN exhibits lower variability than the reference methods. The reduced dispersion observed in convergence time, directional estimation error, and velocity variance suggests greater consistency across different environmental conditions and robot configurations. Compared with Hill Climbing, the proposed controller maintains the gradient-following behavior while incorporating distributed directional cooperation among neighboring modules. Compared with ACO and GA, the proposed controller avoids iterative pheromone updating and population-based search, which contributes to lower variability and faster convergence under the evaluated physical conditions.

Figure 7 presents the distribution of the evaluated metrics considering all experimental executions.

The experimental results demonstrate that the proposed SMG-DN controller improves directional estimation, convergence time, navigation efficiency, and motion stability under heterogeneous soil moisture conditions. These improvements were consistently observed across the evaluated initial conditions, robot configurations, and reference controllers.

## 6 Discussion

The experimental results show that local soil moisture gradients can provide directional information for distributed navigation in modular robotic systems. The statistical analysis performed on the complete experimental dataset, comprising 360 physical executions and approximately $1.5\times 10^{5}$ temporal samples, indicated systematic differences among the evaluated controllers. The one-way ANOVA showed statistically significant differences for convergence time, directional estimation error, navigation efficiency, and velocity variance. These results indicate that the observed differences are not only associated with isolated experimental trials, but with consistent changes in navigation behavior across initial conditions, robot configurations, and repeated physical executions.

The performance differences can be explained by the way each controller transforms environmental information into directional decisions. SMG-DN directly updates the movement direction from local soil moisture variations and obstacle proximity while aggregating directional information from neighboring modules. Hill Climbing uses the same local utility but does not include cooperative aggregation, which explains its lower performance relative to SMG-DN. ACO and GA rely on probabilistic or population-based search mechanisms, which introduce additional variability when the utility values of neighboring candidate directions are similar. Therefore, the lower convergence time and velocity variance observed for SMG-DN are associated with direct gradient-based directional estimation and distributed information exchange rather than with iterative search.

The effect of the local gradient magnitude was also evident across the evaluated initial positions. Initial condition A produced lower convergence times and smaller directional estimation errors, whereas initial condition C produced slower convergence and larger errors. This behavior is expected because stronger moisture gradients generate clearer differences among candidate directions. When the local gradient approaches zero, the directional utility values become closer to each other, reducing the contrast between feasible movement directions. Under these conditions, the robot may exhibit slower directional correction, short oscillatory movements, or temporary exploration around regions with similar moisture values. These cases do not necessarily represent controller failure; rather, they indicate that the available environmental information is insufficient to define a dominant movement direction at that location.

The experimental trials also showed that obstacle interactions can modify the expected gradient-following behavior. When proximity measurements increased in the direction of higher moisture, the obstacle penalty term reduced the directional utility of that candidate movement. In these cases, the robot selected alternative directions that were not always aligned with the maximum moisture increase. This behavior increased convergence time in some trials, but prevented direct motion toward nearby obstacles. Therefore, the navigation behavior results from the interaction between attraction toward higher soil moisture and repulsion generated by obstacle proximity.

Robot configuration also influenced the physical execution of the selected directions. Although the navigation layer computes movement directions from local sensing and neighbor-to-neighbor information exchange, the locomotion layer must transform those directions into physical displacement through the available modular morphology. Chain-type configurations produced a more direct correspondence between the selected direction and the traveling wave generated by the CPG network, which favored smoother displacement and lower velocity variability. Configurations with a larger number of modules increased the spatial distribution of sensing points and provided more information about local moisture variations, but they also introduced additional coordination requirements among oscillators. Branched or more articulated configurations modified the contact pattern with the surface and increased the mechanical effect of lateral modules during direction changes. Therefore, the observed navigation performance was not determined only by the directional estimation algorithm, but also by the ability of each morphology to execute the selected direction through coordinated locomotion. This result justifies evaluating the controller under multiple modular configurations instead of restricting the validation to a single morphology.

The interaction between the navigation layer and the CPG-based locomotion layer is central to the observed behavior. The navigation layer continuously updates the desired movement direction from local environmental information, while the locomotion layer transforms this information into coordinated actuator commands through phase-coupled oscillators. The use of the same locomotion layer for all controllers isolates the effect of the directional estimation strategy. Therefore, the lower velocity variance obtained by SMG-DN can be attributed to more stable directional inputs being supplied to the CPG network, rather than to changes in the locomotion mechanism itself.

The obtained results are related to previous studies on modular robotics, distributed sensing, and CPG-based locomotion. Previous modular robotic approaches have demonstrated distributed communication and physical reconfiguration capabilities, while other works have applied optimization-based methods such as ACO or GA for search and path-selection problems. However, direct numerical comparison with these studies is limited because they generally evaluate different robot platforms, environmental cues, locomotion mechanisms, and performance metrics. The present work differs by evaluating soil moisture gradients as the primary navigation cue and by coupling distributed directional estimation with CPG-based locomotion in physical trials. Therefore, the comparison with prior work is mainly based on methodological scope: the proposed architecture integrates sensing, directional estimation, and locomotion within the same control loop, whereas previous approaches often address these components separately.

Several practical engineering factors may have affected the experimental results. First, the AS7341-based sensing module estimates soil moisture indirectly from infrared reflectance, which can be affected by soil surface texture, illumination, and local reflectance variations. Although calibration was used to convert the sensor response into volumetric water content, measurement noise can reduce the reliability of local gradient estimates. Second, proximity sensing depends on the orientation and range of the VCNL4010 sensors, so obstacles outside the sensing cone may not be represented in the directional utility. Third, inter-module communication and telemetry transmission introduce delays associated with wireless exchange and packet processing. These delays are small relative to the control cycle, but they can affect synchronization when the robot changes direction or interacts with obstacles.

The computation-time analysis indicates that all evaluated controllers completed directional estimation within the 50 ms control cycle. However, the computational cost differed among methods. Hill Climbing required the lowest computation time because it directly selects the maximum local utility. SMG-DN required additional computation due to cooperative aggregation among neighboring modules, but remained below the cost of ACO and GA. ACO and GA required more computation because they include pheromone updating or population-based operations. These results support the real-time feasibility of SMG-DN on the ESP32-WROOM-32E platform while showing that the proposed method maintains lower computational complexity than the iterative optimization baselines.

The experimental validation presents limitations. The experiments were conducted in controlled physical environments and therefore represent a limited range of agricultural field conditions. The study focused on soil moisture as the navigation variable and did not include additional soil properties such as compaction, salinity, or temperature. The calibration model was based on infrared reflectance and may require adaptation for soils with different texture, organic matter, or surface conditions. In addition, the evaluation was limited to the EMeRGe modular platform and the tested configurations. Although the formulation is based on local sensing and neighbor-to-neighbor communication, further experiments are required before generalizing the results to other modular or articulated robotic systems.

Overall, the results indicate that integrating distributed environmental sensing with a CPG-based locomotion layer improves navigation performance under heterogeneous soil moisture conditions. The proposed SMG-DN controller reduced convergence time and directional estimation error while maintaining lower velocity variance than the reference methods. These improvements are explained by the direct use of local gradient information, the aggregation of directional information among neighboring modules, and the continuous transformation of directional estimates into coordinated physical displacement.

## 7 Conclusion

This work presented the Soil-Moisture Gradient Distributed Navigation (SMG-DN) architecture for modular robots operating in heterogeneous soil moisture environments. The proposed approach integrates local environmental sensing, distributed directional estimation, and CPG-based locomotion to generate movement directions from soil moisture gradients without requiring a global map or centralized control.

The experimental evaluation, comprising 360 physical executions under three initial conditions, three robot configurations, and four controllers, showed that SMG-DN improved convergence time, directional estimation accuracy, navigation efficiency, and velocity stability relative to Hill Climbing, ACO, and GA. The comparison with Hill Climbing indicates that the improvement was associated not only with local gradient following, but also with cooperative aggregation of directional information among neighboring modules. The comparison with ACO and GA indicates that direct gradient-based directional estimation reduced the variability introduced by iterative probabilistic or population-based search mechanisms.

The results also showed that navigation performance depends on the spatial structure of the moisture field and on the modular configuration used to execute the selected direction. Stronger local gradients provided clearer directional information, whereas weaker gradients increased uncertainty in movement selection. The computation-time analysis confirmed that SMG-DN remained compatible with the 50 ms control cycle on the ESP32-WROOM-32E platform.

The experimental evidence reported in this study is limited to the EMeRGe platform, the tested modular configurations, and controlled heterogeneous soil moisture conditions. Future work will extend the evaluation to larger agricultural environments, incorporate additional soil and terrain variables, and analyze the effect of communication delays, sensor noise, and adaptive locomotion mechanisms on distributed navigation performance.

## Data Availability

The datasets generated and analyzed during this study are available from the corresponding author upon reasonable request.
