# Correction: Distributed environmental sensing and soil moisture gradient navigation in modular robots

**DOI:** 10.3389/frobt.2026.1987759

**Published:** 2026-09-21

**Authors:** Henry Alberto Hernandez Martinez, Holman Montiel Ariza, Luis Fernando Pedraza Martinez

**Affiliations:** 1 Doctoral Program in Engineering, Faculty of Engineering, Universidad Distrital Francisco José de Caldas, Bogotá, Colombia; 2 Faculty of Technology, Universidad Distrital Francisco José de Caldas, Bogotá, Colombia

**Keywords:** agricultural robotics, autonomous exploration, central pattern generators, chain-type robots, cooperative decision-making, directional estimation, distributed control, environmental monitoring

The author order was incorrect in the published article. The correct author list reads:

Henry Alberto Hernandez Martinez, Holman Montiel Ariza and Luis Fernando Pedraza Martinez.

The original version of this article has been updated.

